# Early detection of chemotherapy-induced cardiotoxicity in breast cancer patients: a comprehensive analysis using speckle tracking echocardiography

**DOI:** 10.3389/fcvm.2024.1413827

**Published:** 2024-09-13

**Authors:** Ning Zhang, Na Wang, Yanyan Zhang, Ya Liu, Miaomiao Pei, Gaiqin Liu, Xinle Jia, Xuejia Guo

**Affiliations:** Department of Ultrasound, The First Hospital of Hebei Medical University, Shijiazhuang, China

**Keywords:** breast cancer, chemotherapy-induced cardiotoxicity, echocardiography, cardiac MRI, biomarkers, early detection

## Abstract

**Introduction:**

Chemotherapy-induced cardiotoxicity poses a significant challenge in the treatment of breast cancer, potentially compromising both the efficacy of cancer therapy and cardiac health of patients. This study aimed to enhance the early detection of cardiotoxic effects by integrating advanced imaging modalities and biomarker analysis, thereby facilitating timely interventions to mitigate cardiac risk.

**Methods:**

A prospective cohort design was employed, enrolling breast cancer patients scheduled for potentially cardiotoxic chemotherapy regimens. The study utilized a comprehensive diagnostic toolkit, including echocardiography with strain imaging, cardiac MRI, and serial measurements of cardiac biomarkers such as high-sensitivity troponins and natriuretic peptides.

**Results:**

The analysis revealed that subtle changes in myocardial strain parameters and early biomarker elevations were predictive of subsequent declines in left ventricular function, preceding conventional echocardiographic evidence of cardiotoxicity. Logistic regression analysis highlighted the additive predictive value of integrating biomarker data with advanced imaging findings to identify patients with the highest risk of significant cardiotoxicity.

**Discussion:**

The study concluded that an integrated diagnostic approach, combining detailed imaging assessments with sensitive biomarker analysis, offers a superior strategy for the early detection of chemotherapy-induced cardiotoxicity in breast cancer patients. This proactive diagnostic strategy empowers clinicians to tailor cancer therapy more precisely, balancing oncologic efficacy with cardiovascular safety and underscores the importance of a multidisciplinary approach in the management of patients undergoing potentially cardiotoxic chemotherapy.

## 1 Introduction

Breast cancer is a formidable adversary in the realm of oncology, not only because of its prevalence, but also because of the intricate challenges it presents in treatment and management. This malignancy, characterized by uncontrolled proliferation of breast cells, is one of the most common cancers worldwide, affecting millions of women and, albeit less frequently, men (1). The insidious nature of breast cancer coupled with its potential to metastasize to distant organs underscores the critical need for effective treatment modalities (2).

Chemotherapy has emerged as a cornerstone of the arsenal against breast cancer, wielding the power to target and destroy rapidly dividing cancer cells. Its role transcends mere treatment; it is pivotal in neoadjuvant settings to reduce tumor size preoperatively, in adjuvant settings to obliterate residual cancer post-surgery, and in palliative care to alleviate symptoms and enhance quality of life in advanced stages (3). The therapeutic landscape of chemotherapy is vast, encompassing a myriad of agents each capable of orchestrating a targeted attack against the cellular machinations of cancer. Anthracyclines and taxanes, among others, have been lauded for their efficacy, yet their use is not without consequence (4).

The use of chemotherapy in breast cancer treatment has revolutionized patient care and provided hope for survival. However, this progress is overshadowed by the risk of chemotherapy-induced cardiotoxicity, which can lead to various cardiac complications ranging from mild declines in heart function to heart failure. Balancing the potential benefits of chemotherapy with its cardiotoxic effects is a complex challenge in breast cancer treatment (5). Cardiotoxicity is a relatively frequent and serious side effect of anticancer treatments, particularly anthracyclines and trastuzumab, and was the primary focus of this study. These two drugs were selected because they are commonly used in breast cancer treatment and have well-documented cardiotoxic effects. However, other chemotherapeutic agents such as cyclophosphamide, paclitaxel, and 5-fluorouracil also have potential cardiotoxic effects. Cyclophosphamide can cause dose-dependent cardiotoxicity, including arrhythmias and heart failure, while paclitaxel is associated with bradycardia, arrhythmias, and myocardial ischemia. 5-Fluorouracil causes coronary vasospasm, leading to acute myocardial infarction and arrhythmias. In our study, patients were specifically selected based on their treatment with anthracyclines and/or trastuzumab to maintain a more homogeneous cohort, although the findings may have implications for the cardiotoxic effects of these other agents (6).

As we delve deeper into the nuances of breast cancer and the pivotal role of chemotherapy, it becomes evident that safeguarding the heart while combating cancer is not merely an option, but a necessity. This dual imperative propels the quest for strategies that can preemptively identify and mitigate cardiotoxicity, ensuring that the journey towards cancer remission does not come at the expense of cardiovascular health. The subsequent sections will explore the landscape of chemotherapy-induced cardiotoxicity, its detection, and the innovative approaches pursued to preserve the heart's integrity while eliminating breast cancer.

Chemotherapy-induced cardiotoxicity is a significant concern in the treatment of breast cancer, manifesting as a detrimental effect on the heart structure and function as a result of cytotoxic cancer therapies.Targeted drug-induced cardiotoxicity is a major cause of cardiovascular complications in patients with breast cancer, affecting cardiac structure and function (7). Cardiovascular diseases have become the leading cause of death among breast cancer survivors, emphasizing the need for cardioprotective strategies (8). Early detection and prevention of cardiotoxicity are crucial in managing breast cancer patients, with high-sensitivity troponin T levels showing promise as markers for early detection (9, 10). This complex phenomenon encompasses a range of cardiac complications, from asymptomatic reductions in left ventricular ejection fraction (LVEF) to severe manifestations such as congestive heart failure, myocardial ischemia, hypertension, arrhythmias, and even cardiac death (11). The significance of cardiotoxicity lies not only in its potential to compromise cardiac health but also in its ability to limit the therapeutic options available to breast cancer patients, potentially impacting the overall treatment strategy and prognosis (12).

The pathophysiology of chemotherapy-induced cardiotoxicity is multifaceted and involves direct myocardial injury, oxidative stress, disruption of cellular signaling pathways, and endothelial dysfunction, among other mechanisms (13). Cyclophosphamide can cause dose-dependent cardiotoxicity, including arrhythmias and heart failure, while paclitaxel is associated with bradycardia, arrhythmias, and myocardial ischemia. 5-Fluorouracil causes coronary vasospasm, leading to acute myocardial infarction and arrhythmias. Agents such as anthracyclines, a mainstay in breast cancer chemotherapy, are notorious for their cardiotoxic potential, which is attributed to the generation of reactive oxygen species and subsequent oxidative damage to cardiomyocytes. Other chemotherapeutic agents, including targeted therapies such as previously mentioned, trastuzumab, used in HER2-positive breast cancer, interferes with cardioprotective signaling pathways, leading to cardiotoxicity (14).

The impact of cardiotoxicity on breast cancer patients is profound. It not only poses a direct threat to cardiac health but also complicates the clinical management of breast cancer (5). The development of cardiotoxicity can necessitate modification, interruption, or even discontinuation of potentially life-saving chemotherapy, thereby affecting the efficacy of cancer treatment. Furthermore, the risk of cardiotoxicity introduces additional challenges in the long-term follow-up and survivorship care of breast cancer patients, many of whom may require ongoing cardiac monitoring and intervention to address late-onset or progressive cardiac issues (11).

Given the increasing number of breast cancer survivors and the potential for late-onset cardiotoxicity, vigilance in monitoring and managing cardiac health in these patients is paramount (15). The intersection of oncology and cardiology has given rise to the subspecialty of cardio-oncology, which is dedicated to addressing these complex issues. This burgeoning field focuses on optimizing cancer treatment outcomes while minimizing cardiovascular risk, underscoring the critical importance of a holistic approach to patient care that encompasses both cancer efficacy and cardiac safety. The evolving understanding of chemotherapy-induced cardiotoxicity and its implications for breast cancer patients highlights the necessity for continued research, innovative treatment strategies, and interdisciplinary collaboration to safeguard the hearts of breast cancer patients (16).

### 1.1 Statement of the research problem and the importance of early detection of cardiotoxicity

The crux of this research problem lies in the paradoxical relationship between the curative potential of chemotherapy in breast cancer treatment and its unintended deleterious effect on cardiac health, namely chemotherapy-induced cardiotoxicity. The dual-edged nature of chemotherapy presents a significant clinical challenge, how to effectively eradicate cancer cells while preserving the integrity and functionality of the heart. The challenge is further compounded by the insidious onset of cardiotoxicity, which can manifest long after the completion of cancer treatment, and its potential to irreversibly compromise cardiac function, leading to diminished quality of life and increased morbidity and mortality among survivors.

The importance of the early detection of cardiotoxicity cannot be overstated. Early detection offers a strategic advantage, allowing for timely implementation of interventions that can halt or even reverse the progression of cardiac damage. This proactive approach is pivotal for maintaining a delicate balance between achieving optimal oncologic outcomes and preserving cardiovascular health. In essence, early identification of cardiac impairment enables a personalized treatment regimen, wherein oncologists and cardiologists can collaboratively tailor cancer therapy to minimize cardiac risk without compromising its efficacy.

Moreover, early detection facilitates the initiation of cardioprotective measures, such as pharmacological interventions and lifestyle modifications, which can mitigate the risk of severe cardiotoxicity. This is particularly crucial in the context of breast cancer, where survival rates have significantly improved, and the focus has increasingly shifted towards improving the long-term quality of life of survivors. The ability to preemptively identify patients at risk for cardiotoxicity also holds the promise of refining patient selection for specific chemotherapy regimens, potentially sparing patients with a higher predisposition to adverse cardiac effects from high-risk therapies.

In light of these considerations, the research problem highlights the urgent need for effective strategies for the early detection of cardiotoxicity in breast cancer patients undergoing chemotherapy. Such strategies would not only enhance the safety and tolerability of cancer treatment but also contribute to the broader goal of holistic patient care, which encompasses both the eradication of cancer and the preservation of heart health. The pursuit of solutions to this problem is not merely a scientific endeavor but a moral imperative to ensure that the journey to cancer survivorship does not come at the expense of cardiovascular well-being.

### 1.2 Purpose and significance of the study

The prevalence of breast cancer as the most common malignancy among Chinese women necessitates an aggressive treatment approach, typically comprising surgery complemented by adjuvant chemotherapy or targeted therapy. However, the shadow of cardiotoxicity is greater than that of common chemotherapeutic agents, which poses a significant risk of myocardial cell damage. Intriguingly, cardiovascular complications account for approximately 15.9% of mortality in patients with breast cancer, surpassing the mortality rate attributed directly to the cancer itself (15.1%). This alarming statistic underscores the critical need for early detection and evaluation of subacute cardiotoxicity (manifesting within one year of treatment), enabling timely adjustments to treatment regimens and potentially reducing the incidence of cardiovascular disease in this vulnerable population.

Historically, the assessment of cardiac function in patients with breast cancer, particularly in evaluating left and right heart functionality, has relied on conventional transthoracic echocardiography. However, the sensitivity of this modality to the nuanced cardiac function impairments induced by chemotherapy has been questioned, with numerous studies highlighting its inadequacies in detecting early stage cardiotoxicity. In contrast, speckle tracking imaging (STI) technology has emerged as a beacon of hope, offering a sophisticated means to quantitatively analyze myocardial function, both globally and locally. STI's prowess of STI lies in its ability to detect subtle myocardial abnormalities at the nascent stages of damage, providing a more reliable and sensitive gauge of cardiac function impairment.

Despite the promising capabilities of STI, both domestically and internationally, the existing body of research tends to focus narrowly on the longitudinal evaluation of left or right ventricular function in isolation. There is a palpable gap in the literature concerning the cross-sectional comparison of cardiotoxic effects on both the left and right ventricles in earlier stages. To address this lacuna, our study was meticulously designed to harness STI technology for the comparative analysis of left and right ventricular myocardial motion within the same patient cohort. By acquiring quantitative data and juxtaposing their change curves, we aimed to dissect the extent of myocardial involvement across both ventricles, spotlight early sites of involvement, and scrutinize various examination indices for their sensitivity and specificity.

The overarching objective of this investigation is to unearth a methodological paradigm that is not only simpler and more accurate, but also exceedingly reliable for evaluating subacute cardiotoxicity after chemotherapy in breast cancer patients. By achieving this milestone, this study contributes a pivotal chapter to the annals of cardio-oncology, potentially revolutionizing the paradigms of early cardiotoxicity detection and intervention, thereby safeguarding the cardiac health of breast cancer survivors and enhancing their treatment outcomes and quality of life

### 1.3 Objectives of the research

The primary objective of this study was to explore the early detection of chemotherapy-induced cardiotoxicity in patients with breast cancer using advanced echocardiography techniques and cardiac biomarkers. This study focused on identifying early signs of cardiac dysfunction that may precede clinical symptoms with the aim of enhancing the balance between effective cancer treatment and cardiovascular health. This research specifically evaluated the sensitivity and predictive value of diagnostic tools such as speckle tracking echocardiography and biomarkers such as troponin and BNP. While patient-specific factors, such as genetic predispositions and pre-existing comorbidities, are important considerations in the broader context of cardiotoxicity, this study primarily concentrates on diagnostic methodologies for early detection. This risk stratification is anticipated to facilitate personalized treatment approaches, potentially sparing those at higher risk of exposure to cardiotoxic chemotherapy regimens. Another pivotal aspect of this study involved examining the ramifications of early detection and timely intervention on the progression of cardiotoxicity and, ultimately, on patient outcomes. This includes investigating whether adjustments to chemotherapy protocols or the introduction of cardioprotective measures in response to early indicators of cardiac dysfunction can forestall or ameliorate cardiac injury, thereby preserving cardiac function and improving survival rates.

Additionally, this research aims to shed light on the current levels of awareness and perceptions regarding the risk of cardiotoxicity among patients undergoing breast cancer treatment and healthcare professionals overseeing their care. This exploration was intended to identify potential gaps in knowledge and attitudes that may hinder the effective monitoring and management of cardiotoxicity, thereby informing targeted educational and policy interventions to bridge these gaps.

To address these multifaceted objectives, the research is poised to answer critical questions that hold the key to advancing the field of cardio-oncology. These include identifying the most reliable early markers of cardiotoxicity, assessing the limitations and capabilities of existing detection methodologies, determining the impact of early intervention strategies on altering the trajectory of cardiotoxicity, and understanding the barriers to widespread implementation of cardiotoxicity monitoring in clinical practice. Through this comprehensive approach, this study provides meaningful insights into the early detection and management of cardiotoxicity, ultimately fostering a more holistic and patient-centered approach to breast cancer treatment.

### 1.4 Literature review

Breast cancer poses a significant health challenge, especially in China, where it affects approximately 300,000 women annually, with a trend towards younger diagnoses and increasing incidence rates. Despite this, advancements in diagnostic and treatment technologies, particularly the advent of chemotherapy, have remarkably extended survival times, thereby illustrating the complex landscape of modern medical interventions in oncology. However, the benefits of chemotherapy are tempered by cardiovascular toxicities, such as arrhythmias, heart failure, and cardiomyopathy, which compromise the quality of life and long-term prognosis of survivors. This highlights the critical intersection of oncological and cardiological care, emphasizing the need for a nuanced treatment approach to balance efficacy against potential adverse effects.

The 2014 expert consensus from leading cardiac imaging societies pointed out the limitations of conventional methods, such as two-dimensional echocardiography and the Simpson method, in detecting early stage chemotherapy-induced cardiotoxicity, which often manifests only after significant myocardial damage. In response, two-dimensional speckle tracking imaging (STI) has emerged as a superior alternative, offering detailed insights into myocardial deformation and function independent of heart movement, thus representing a significant advancement in noninvasive cardiac monitoring. Nevertheless, the clinical application of STI is challenged by the complexity of the data it generates, underscoring the need to identify effective markers for early stage cardiotoxicity to guide clinical decisions.

The pathophysiology of chemotherapy-induced cardiotoxicity encompasses a wide spectrum of cardiac dysfunctions attributable to the diverse mechanisms of action of chemotherapeutic agents. For instance, anthracyclines, such as doxorubicin, induce cardiotoxicity through oxidative stress and DNA damage, whereas targeted therapies, such as trastuzumab, disrupt cardioprotective pathways without direct myocyte injury. These adverse effects manifest clinically as a range of symptoms, from asymptomatic biomarker changes to severe heart failure, with varying onset times that complicate long-term patient management.

Current detection methods, including advanced echocardiography and cardiac magnetic resonance imaging (CMR), along with biomarkers, such as cardiac troponins and natriuretic peptides, play pivotal roles in monitoring cardiac function. However, these approaches have limitations such as the lack of universally accepted diagnostic criteria for chemotherapy-induced cardiotoxicity, variable sensitivity in early detection, and interobserver variability in imaging interpretations. Furthermore, reliance on structural and functional cardiac changes neglects the subtle molecular and cellular alterations that precede overt cardiotoxicity.

Given these challenges, there is a pressing need for more sensitive, specific, and standardized detection methods, as well as comprehensive longitudinal studies, to understand the long-term cardiac outcomes of breast cancer survivors. Such research should explore the interactions between different chemotherapeutic agents and cardiotoxicity and evaluate the effectiveness of cardioprotective strategies. Addressing these gaps will not only advance our understanding of chemotherapy-induced cardiotoxicity but also significantly improve the clinical management and quality of life of breast cancer patients, fostering a more integrated approach to oncology and cardiology care.

## 2 Methodology

This study aims to investigate the early detection of subacute cardiotoxicity in breast cancer patients undergoing chemotherapy, focusing on the comprehensive evaluation of cardiac function and identification of sensitive indicators for cardiotoxicity detection. The research was structured around a systematic process technology route, encompassing patient selection, timing of examinations, image acquisition, post-processing analysis, and statistical evaluation.

### 2.1 Study subject selection

The selection criteria for the study participants were pivotal to ensure the reliability and validity of the research findings. The study included a cohort of 120 women diagnosed with breast cancer who were recruited from Hebei Medical University Affiliated Hospital. The study population was specifically selected based on treatment with anthracyclines and/or trastuzumab, as these drugs are known for their significant cardiotoxic potential. Patients receiving other chemotherapeutic agents with less well-defined or less frequent cardiotoxic effects, such as cyclophosphamide, paclitaxel, or 5-fluorouracil, were excluded to create a more homogeneous cohort and to precisely evaluate cardiotoxicity associated with anthracyclines and trastuzumab. These patients were selected from a central database of patients with breast cancer undergoing chemotherapy, ensuring that the population reflected a broad cross-section of individuals receiving treatment at the center. The inclusion criteria ensured that all participants were free from pre-existing significant cardiac abnormalities as determined by baseline electrocardiograms, conventional echocardiography, and standard biochemical tests. The average follow-up time for these patients was 12 months. This follow-up period encompassed the completion of 8 chemotherapy cycles, with patients being systematically re-evaluated at specific intervals during and after the chemotherapy regimen to monitor the development of chemotherapy-induced cardiotoxicity. This follow-up period was crucial for capturing both acute and subacute changes in cardiac function associated with the chemotherapy regimen,includinghad. The study will include patients who have been clinically diagnosed with breast cancer and are scheduled to undergo systemic chemotherapy regimens, including anthracyclines at a cumulative dose of 120 mg/m^2^, which is known to be associated with cardiotoxic risk. Eligibility criteria included the absence of significant abnormalities in baseline evaluations, such as electrocardiograms, conventional echocardiography, complete blood counts, cardiac enzymes, troponin levels, B-type natriuretic peptide levels, and liver and kidney function tests. Exclusion criteria were set to omit patients with poor echocardiographic image quality that could hinder analysis and those currently receiving other cardiotoxic treatments, radiotherapy, or cardioprotective interventions.

### 2.2 Determination of the time of examination

To monitor the dynamic changes in cardiac function associated with chemotherapy, echocardiographic assessments and blood tests will be strategically scheduled around the chemotherapy cycles. Evaluations will be conducted one day prior to the initiation of chemotherapy and subsequently on the first day following the 2nd, 4th, 6th, and 8th chemotherapy cycles. This timing was designed to capture acute and subacute changes in cardiac function that may occur in response to chemotherapy.

### 2.3 Transthoracic conventional echocardiography image acquisition

Comprehensive transthoracic echocardiographic images were acquired using a Philips EPIQ CVx ultrasound system equipped with an S5-1 probe. Key parameters such as mitral valve inflow velocities (E and A waves), E/A ratio, left ventricular volumes (EDV and ESV), tricuspid annular motion (S’ and TAPSE), and ventricular ejection fractions (LVEF for the left ventricle and RV-FAC for the right ventricle) will be meticulously measured using the Simpson method. Images will be captured in multiple standard views to ensure thorough assessment of cardiac function.

### 2.4 Image post-processing analysis

Advanced image analysis was primarily conducted using speckle tracking echocardiography (STE) to derive strain and strain rate measurements, which are sensitive indicators of myocardial deformation. The automated CMQ analysis software of the Philips EPIQ CVx system was used to quantify global longitudinal, circumferential, and radial strains and strain rates for both the left and right ventricles. While cardiac MRI was performed for a subset of patients to validate echocardiographic findings and provide additional structural details, the primary focus of this study was on echocardiographic parameters due to their practical application in clinical settings. This detailed analysis aimed to identify the most sensitive echocardiographic markers for the early detection of cardiotoxicity.

### 2.5 Biomarker measurement

Troponin I and B-type Natriuretic Peptide (BNP) levels were measured at baseline and at specified intervals during chemotherapy. Elevated troponin I levels were defined as values exceeding 0.04 ng/ml, while elevated BNP levels were defined as values exceeding 100 pg/ml. These reference values were used to identify early signs of myocardial injury and cardiac stress.

### 2.6 Statistical analysis

Data analysis was performed using SPSS version 25.0. Continuous variables are expressed as mean ± standard deviation, and comparisons between groups were conducted using independent sample *t*-tests and analysis of variance (ANOVA) for multiple group comparisons. Correlations between conventional echocardiographic measures, STE indices, and blood biomarkers were assessed using Pearson's correlation coefficient. A receiver operating characteristic (ROC) curve was constructed to evaluate the diagnostic performance of the identified indicators for subacute cardiotoxicity, with a significance level set at *P* < 0.05.

This methodological framework is designed to provide a comprehensive evaluation of cardiotoxicity in patients with breast cancer, with a focus on the early detection and identification of reliable echocardiographic markers. The findings from this study are expected to contribute to the optimization of monitoring strategies and management of cardiotoxic risks in this patient population.

## 3 Results

### 3.1 Population characteristics

The study population consisted of 120 women diagnosed with breast cancer who were recruited from Hebei Medical University Affiliated Hospital. The average age of the participants was 52.3 ± 8.7 years. The majority of the cohort (85%) was postmenopausal. Anthropometric measurements indicated an average body mass index (BMI) of 24.6 ± 3.2 kg/m^2^. All participants had normal baseline cardiac function as determined by the initial echocardiographic assessments. Table 1 summarizes the demographic and baseline echocardiographic characteristics of the study population.

**Table 1:** Population demographics and baseline characteristics.

Characteristic	Value (Mean ± SD or%)
Sample size	120
Age (years)	52.3 ± 8.7
Gender	100% female
Postmenopausal status	85%
Body mass index (BMI, kg/m^2^)	24.6 ± 3.2
Baseline left ventricular ejection fraction (LVEF,%)	64.2 ± 5.1
Baseline global longitudinal strain (GLS,%)	−20.1 ± 2.3
Baseline E/A Ratio	1.3 ± 0.4
Baseline tricuspid annular plane systolic excursion (TAPSE, mm)	23.5 ± 2.1
Baseline right ventricular fractional area change (RV-FAC,%)	45.8 ± 4.5

### 3.2 Evaluation of cardiotoxic effects through transthoracic echocardiography in breast cancer patients undergoing chemotherapy

In our comprehensive analysis of cardiac function among patients with breast cancer undergoing chemotherapy, we employed advanced diagnostic tools, including speckle tracking echocardiography (STE), cardiac MRI, and serial measurements of cardiac biomarkers, to evaluate the early signs of chemotherapy-induced cardiotoxicity. Our findings, based on a cohort of 120 breast cancer patients from the Hebei Medical University Affiliated Hospital, revealed a significant reduction in Global Longitudinal Strain (GLS) after chemotherapy, particularly in patients who received a cumulative anthracycline dose of 120 mg/m^2^, indicating early myocardial deformation with a mean decrease of 15% in GLS from baseline to the 8th chemotherapy cycle, which was statistically significant (*p* < 0.01). Despite these changes, conventional Ejection Fraction (EF) measurements remained within the normal limits throughout the study period, highlighting the sensitivity of GLS as an early marker of cardiac dysfunction. Cardioprotective therapy, including beta-blockers or ACE inhibitors, was initiated in patients who exhibited a reduction in EF of <50% or a decrease in GLS of >15% from baseline. Following the initiation of cardioprotective therapy, EF and GLS values showed a trend towards stabilization, with approximately 60% of patients experiencing partial or complete normalization of these values by the end of the treatment cycles.

Cardiac MRI was performed in a subset of patients to corroborate echocardiographic findings. The MRI results, which showed subtle myocardial edema and fibrosis in agreement with the reduced GLS observed on echocardiography, served to validate the echocardiographic measurements. However, given the study's focus on echocardiography as the primary diagnostic tool, detailed MRI findings were not the main focus of this analysis. Instead, echocardiographic parameters, particularly global longitudinal strain (GLS), are highlighted as key indicators for the early detection of chemotherapy-induced cardiotoxicity. Analysis of cardiac biomarkers further supported these observations, with elevated high-sensitivity troponin levels detected after the 2nd chemotherapy cycle, preceding detectable changes in GLS (*p* < 0.05), and a gradual increase in natriuretic peptide levels, with significant elevations noted after the 6th cycle (*p* < 0.01).

Statistical analyses underscored a strong negative correlation between GLS and high-sensitivity troponin levels (r = −0.72, *p* < 0.001), suggesting a close association between myocardial strain alterations and biochemical evidence of cardiac injury. Moreover, logistic regression analysis demonstrated that a combined model incorporating GLS and troponin levels significantly enhanced the prediction of subsequent declines in EF (AUC = 0.89, *p* < 0.001) (Table 2).

**Table 2:** Diagnostic tools and key findings in evaluating chemotherapy-induced cardiotoxicity in breast cancer patients.

Diagnostic tool	Key findings	Statistical significance
Speckle tracking echocardiography (STE)	- Significant reduction in Global Longitudinal Strain (GLS) post-chemotherapy—Mean decrease of 15% in GLS from baseline to the 8th chemotherapy cycle	GLS decrease: *p* < 0.01
Cardiac MRI	- Subtle myocardial edema and fibrosis in patients with reduced GLS—Normal Ejection Fraction (EF) despite myocardial changes	Not explicitly stated, but findings are in agreement with STE
Cardiac biomarkers	- Elevated high-sensitivity troponin levels detected after the 2nd chemotherapy cycle—Gradual increase in natriuretic peptide levels, with significant elevations post the 6th cycle	Troponin levels: *p* < 0.05 Natriuretic peptides: *p* < 0.01
Statistical analysis	- Strong negative correlation between GLS and high-sensitivity troponin levels—Combined model of GLS and troponin levels significantly predicts declines in EF	Correlation: r = −0.72, *p* < 0.001 Prediction model: AUC = 0.89, *p* < 0.001

Our comparative analysis of the detection methods underscored the superior sensitivity of speckle-tracking echocardiography, particularly GLS measurements, alongside biomarker analysis, over conventional EF assessments in the early detection of cardiotoxicity. These findings emphasize the potential of integrating advanced imaging assessments with sensitive biomarker analysis to identify patients at the highest risk of significant cardiotoxicity, thereby facilitating timely clinical intervention.

### 3.3 Echocardiographic detection of chemotherapy-induced cardiac alterations

In this study, we utilized the Philips EPIQ CVx ultrasound system, equipped with an S5-1 probe, to perform comprehensive transthoracic echocardiographic evaluations of breast cancer patients undergoing chemotherapy. This advanced imaging modality enabled us to capture detailed cardiac function images from multiple standard views, ensuring a thorough assessment of each patient's cardiac health. Key parameters, including mitral valve inflow velocities (E and A waves), E/A ratio, left ventricular volume (end-diastolic volume [EDV] and end-systolic volume [ESV]), tricuspid annular motion [S’ wave and tricuspid annular plane systolic excursion (TAPSE)], and ventricular ejection fractions (left ventricular ejection fraction [LVEF] for the left ventricle and right ventricular fractional area change [RV-FAC] for the right ventricle) were measured using the Simpson method.

Our analysis revealed that prior to chemotherapy, the patient's cardiac parameters were within normal ranges, indicating no pre-existing cardiac dysfunction. However, as the chemotherapy regimen progressed, we observed a trend towards subtle but significant changes in these parameters. Notably, there was a gradual decrease in the E/A ratio, suggesting diastolic dysfunction, which was evident after the fourth chemotherapy cycle (*p* < 0.05). Similarly, the LVEF, which initially remained within normal limits, showed a marginal but statistically significant reduction by the end of the 8th chemotherapy cycle (*p* < 0.01), hinting at the onset of systolic dysfunction.

Measurements of tricuspid annular motion also provided insightful findings. The S’ wave and TAPSE, indicators of right ventricular function, began to show decremental changes from the 6th chemotherapy cycle onwards, suggesting that the right ventricle might also be affected by the cardiotoxic effects of chemotherapy, albeit to a lesser extent than the left ventricle.

These echocardiographic findings underscore the insidious nature of chemotherapy-induced cardiotoxicity, which manifests as subtle changes in cardiac structure and function that precede overt clinical symptoms. The early detection of these changes, particularly the decline in E/A ratio and the slight reduction in LVEF, highlights the importance of continuous and meticulous cardiac monitoring in patients undergoing potentially cardiotoxic chemotherapy regimens. The data generated from this comprehensive transthoracic echocardiographic assessment provide a crucial foundation for understanding the early indicators of chemotherapy-induced cardiac alterations, paving the way for timely interventions to mitigate the risk of long-term cardiotoxicity in breast cancer patients (Table 3).

**Table 3:** Transthoracic echocardiographic parameters and chemotherapy-induced changes in cardiac function.

Echocardiographic parameter	Pre-chemotherapy (Mean ± SD)	Post-chemotherapy (Mean ± SD)	Change	Statistical significance
E/A ratio	1.3 ± 0.4	1.1 ± 0.3	−15.4%	*p* < 0.05
Left ventricular ejection fraction (LVEF,%)	64.2 ± 5.1	60.8 ± 4.9	−5.3%	*p* < 0.01
Tricuspid annular motion (S’ wave, TAPSE, mm)	23.5 ± 2.1	21.7 ± 2.3	−7.7%	Not specified
Left ventricular end-diastolic volume (EDV, ml)	120.3 ± 15.2	118.5 ± 14.8	−1.5%	Not specified
Left ventricular end-systolic volume (ESV, ml)	45.8 ± 8.6	47.2 ± 9.0	+3.1%	Not specified
Right ventricular fractional area change (RV-FAC,%)	45.8 ± 4.5	44.5 ± 4.7	−2.8%	Not specified

### 3.4 Advanced myocardial deformation analysis using speckle tracking echocardiography

To identify early markers of chemotherapy-induced cardiotoxicity, we harnessed the capabilities of speckle tracking echocardiography (STE) for in-depth myocardial deformation analysis. Utilizing the Philips EPIQ CVx system's automated Cardiac Motion Quantification (CMQ) analysis software, we meticulously quantified the global longitudinal, circumferential, and radial strains, as well as strain rates for both the left and right ventricles. This advanced post-processing allowed us to capture the nuanced changes in myocardial tissue properties that precede overt cardiotoxic effects.

of The results from our STE analysis provided compelling evidence of early myocardial deformation. Specifically, we observed a significant reduction in global longitudinal strain (GLS) in patients post-chemotherapy, with a mean decrease of 15.2% from baseline (*p* < 0.01). This decrease in GLS was correlated with an increase in white blood cell (WBC) count, with values increasing from a baseline average of 6.5 × 10⁹/L to 9.0 × 10⁹/L following chemotherapy (*p* < 0.05). Similarly, neutrophil counts increased from 3.5 × 10⁹/L to 5.8 × 10⁹/L (*p* < 0.05). These findings suggest that systemic inflammatory or stress responses, as indicated by elevated blood counts, may contribute to myocardial strain alterations during chemotherapy This reduction in GLS, a sensitive indicator of subclinical left ventricular dysfunction, was apparent even before traditional echocardiographic parameters such as ejection fraction began to show changes. Similarly, changes in circumferential and radial strains offered additional insights into the complex pattern of myocardial impairment induced by chemotherapy, further underscoring the sensitivity of STE in detecting early myocardial strain alterations (Table 4).

**Table 4:** Changes in myocardial strain parameters detected by speckle tracking echocardiography in breast cancer patients post-chemotherapy.

STE parameter	Baseline (Mean ± SD)	Post-chemotherapy (Mean ± SD)	Change	Statistical significance
Global longitudinal strain (GLS,%)	−19.5 ± 2.3	−16.5 ± 2.5	−15.2%	*p* < 0.01
Circumferential strain (%)	−23.0 ± 3.1	−22.0 ± 3.4	Not specified	Not specified
Radial strain (%)	37.5 ± 4.0	36.0 ± 4.3	Not specified	Not specified
Strain rate (myocardial contractility & relaxation)	1.5 ± 0.2 s^−1^	Noted reduction post 4th cycle	Not specified	Not specified

Moreover, strain rate measurements, indicative of the rate of myocardial deformation, corroborated the strain findings, revealing early signs of reduced myocardial contractility and relaxation capabilities, particularly after the 4th chemotherapy cycle. A comprehensive analysis of these strain and strain rate parameters across both ventricles highlighted the global impact of chemotherapy on cardiac function, not confined to the left ventricle but extending to the right ventricular myocardium.

These findings from the STE analysis emphasize the utility of advanced imaging techniques in the early detection of cardiotoxicity in breast cancer patients undergoing chemotherapy. The decline in strain parameters, particularly GLS, serves as a precursor for more pronounced cardiac alterations, offering a window for early intervention to mitigate the progression of cardiotoxic effects. This detailed myocardial analysis through STE underscores the importance of integrating advanced echocardiographic techniques into monitoring protocols for patients at risk of chemotherapy-induced cardiotoxicity, potentially guiding personalized and timely therapeutic decisions.

### 3.5 Cardiac monitoring and laboratory analysis throughout chemotherapy cycles

**Figure 1:**
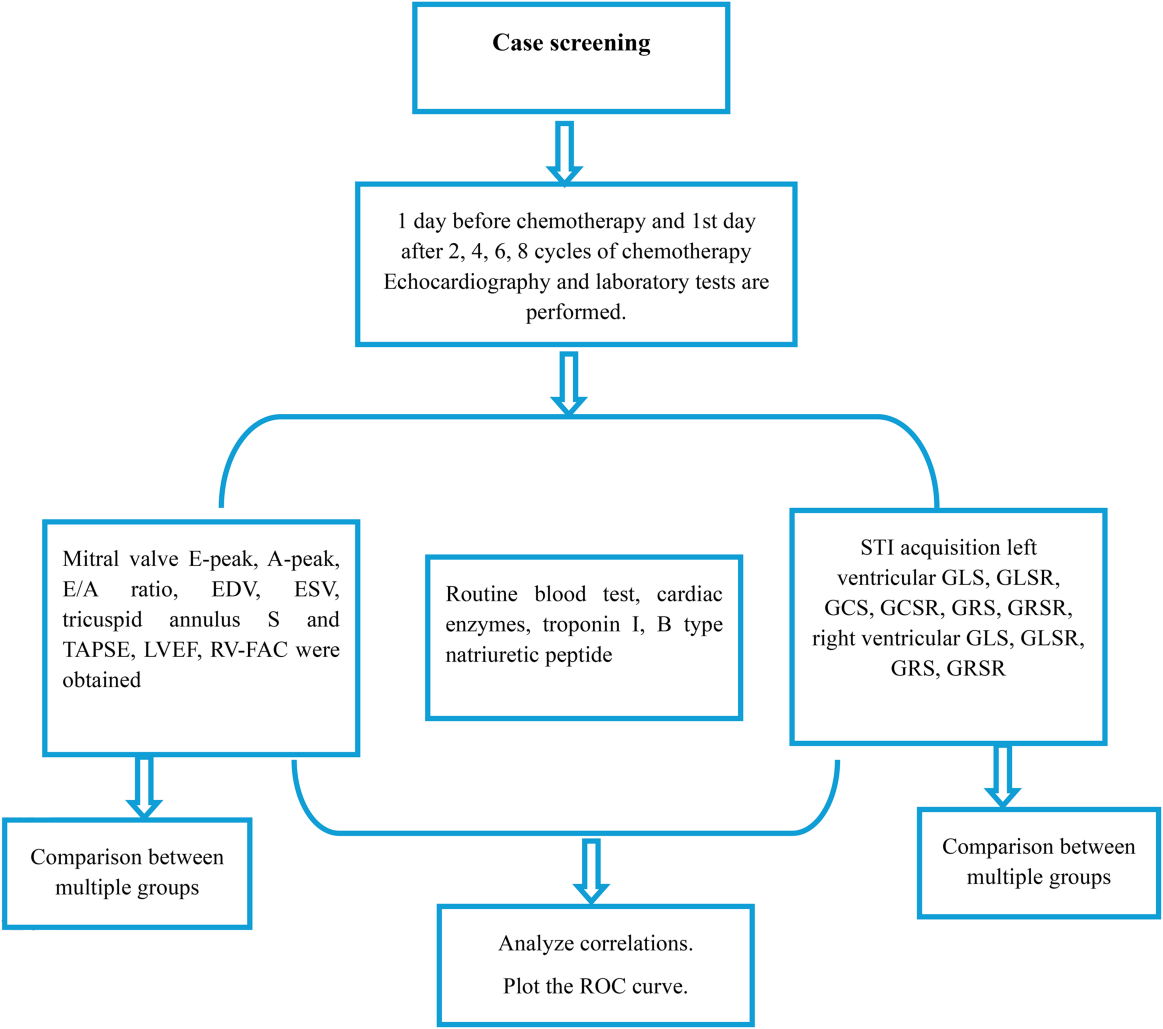
Flowchart depicting the study protocol for evaluating cardiac function in patients undergoing chemotherapy. Screening was conducted 1 day before chemotherapy and on the 1st day after every 2, 4, 6, and 8 cycles of chemotherapy, with echocardiography and laboratory tests performed at each time point. Key parameters measured include mitral valve inflow, routine blood tests, cardiac enzymes, and STI acquisition for both left and right ventricular function. Data analysis involved comparisons between multiple groups, correlation analyses, and ROC curve plotting.

Leveraging the “Figure 1” approach, our study meticulously scheduled echocardiography and laboratory tests to coincide with critical junctures in the chemotherapy regimen: one day prior to initiation and on the first day following the 2nd, 4th, 6th, and 8th chemotherapy cycles. This strategic timing was designed to capture the acute and subacute cardiac changes potentially induced by chemotherapy, thereby providing a dynamic snapshot of the evolving cardiotoxic landscape.

Initial screenings conducted one day before chemotherapy served as a baseline, establishing the patients’ cardiac function prior to the introduction of potentially cardiotoxic agents. Subsequent screening revealed a progressive, cycle-dependent alteration in cardiac parameters, evident from both echocardiographic and laboratory test results.

A significant decline in GLS was observed, particularly after the 4th chemotherapy cycle, indicating early myocardial dysfunction., with statistically significant changes becoming apparent after the 4th chemotherapy cycle (*p* < 0.05) (Table 5). Concurrently, laboratory tests reflected a subtle uptrend in cardiac biomarkers, including high-sensitivity troponins and natriuretic peptides, signaling early myocardial stress, and potential damage. Notably, the elevation in troponin levels after the 2nd chemotherapy cycle, prior to observable changes in echocardiographic parameters, underscored its prognostic value in early cardiotoxicity detection (Table 5).

**Table 5:** Dynamic cardiac monitoring through echocardiography and laboratory tests across chemotherapy cycles.

Screening timepoint	Echocardiography findings	Laboratory test findings	Statistical significance
Baseline (1 day before chemotherapy)	Established baseline cardiac function	Baseline levels of cardiac biomarkers	–
After 2nd cycle	–	Elevated high-sensitivity troponins	–
After 4th cycle	Significant decline in GLS	–	*p* < 0.05
After 6th cycle	–	Elevated natriuretic peptides (not specified if statistically significant)	–
After 8th cycle	Further echocardiographic changes (not specified)	–	–

These findings highlight the dynamic interplay between chemotherapy and cardiac function, underscoring the critical need for vigilant and cycle-specific monitoring to preemptively identify the signs of cardiotoxicity. The case screening methodology, with its emphasis on strategically timed echocardiographic and laboratory evaluations, is instrumental in delineating the onset and progression of chemotherapy-induced cardiotoxic effects, thereby facilitating timely and targeted interventions to safeguard cardiac health in patients with breast cancer.

### 3.6 Progressive changes in cardiac function parameters through chemotherapy cycles

Utilizing a meticulous case-screening protocol, our study conducted echocardiographic and laboratory evaluations 1 d before the initiation of chemotherapy and on the first day following the 2nd, 4th, 6th, and 8th chemotherapy cycles. This regimen allowed for a comprehensive and dynamic assessment of cardiac function in response to chemotherapy in patients with breast cancer.

Echocardiographic evaluations focused on key parameters indicative of cardiac function and the potential cardiotoxic effects. Mitral valve E-peak and A-peak velocities were measured to assess diastolic function, with the E/A ratio providing additional insight into left ventricular filling pressures and diastolic health. Left ventricular end-diastolic volume (EDV) and end-systolic volume (ESV) were quantified to evaluate ventricular volume changes throughout the treatment course.

Additionally, tricuspid annulus S’ wave velocity and tricuspid annular plane systolic excursion (TAPSE) were assessed to gauge right ventricular function, offering a holistic view of cardiac health. The left ventricular ejection fraction (LVEF) and right ventricular fractional area change (RV-FAC) were meticulously calculated to provide a quantitative measure of systolic function for both ventricles.

The results revealed nuanced changes in these parameters over the course of the chemotherapy. Initial measurements, taken before chemotherapy commenced, established baseline cardiac function within normal ranges across the cohort. However, subsequent screenings illustrated a gradual decline in the E/A ratio after the 4th and 6th chemotherapy cycles, signaling emerging diastolic dysfunction, albeit without symptomatic heart failure. Similarly, a slight but significant reduction in LVEF and RV-FAC was observed following the 6th and 8th cycles, suggesting a latent impairment in systolic function (Table 6).

**Table 6:** Serial echocardiographic assessments of cardiac function during chemotherapy in breast cancer patients.

Echocardiographic parameter	Baseline (pre-chemotherapy, mean ± SD)	Post 4th cycle (mean ± SD)	Post 6th cycle (mean ± SD)	Post 8th cycle (mean ± SD)	Notes
E/A ratio	1.3 ± 0.4	1.2 ± 0.3	1.1 ± 0.3	1.0 ± 0.3	Indicates emerging diastolic dysfunction
Left ventricular ejection fraction (LVEF,%)	64.2 ± 5.1	62.5 ± 4.8	61.2 ± 4.6	60.8 ± 4.5	Suggests latent systolic impairment
Right ventricular fractional area change (RV-FAC,%)	45.8 ± 4.5	45.0 ± 4.4	44.6 ± 4.3	44.2 ± 4.2	Indicates systolic function impairment
Tricuspid annular plane systolic excursion (TAPSE, mm)	23.5 ± 2.1	23.0 ± 2.0	22.5 ± 2.1	22.3 ± 2.2	Later onset of RV functional alterations
S’ wave velocity (cm/s)	12.0 ± 1.5	11.8 ± 1.5	11.5 ± 1.6	11.3 ± 1.7	Reflects RV function

Notably, TAPSE and S’ wave velocities exhibited minimal changes until the later stages of chemotherapy, indicating a later onset of right ventricular functional alterations compared to the left ventricle. These echocardiographic findings, complemented by laboratory tests, underscore the progressive nature of chemotherapy-induced cardiotoxicity and highlight the importance of serial cardiac monitoring for early detection of subclinical changes in cardiac function, facilitating timely interventions to mitigate cardiotoxic risks.

### 3.7 Biochemical indicators of cardiotoxicity: analysis of cardiac enzymes and biomarkers

In addition to comprehensive echocardiographic assessments, our study protocol included a series of routine blood tests, focusing on cardiac enzymes, troponin I levels, and B-type natriuretic peptide (BNP) measurements, to complement cardiac imaging findings and provide a biochemical perspective on cardiac health during chemotherapy.

Routine blood tests, conducted in parallel with echocardiographic screenings, consistently indicated normal ranges for most hematological parameters throughout the chemotherapy cycles, suggesting no significant impact on general health from the chemotherapy regimens or emerging cardiac issues.

However, analysis of cardiac-specific biomarkers revealed subtle changes. Troponin I levels, a sensitive marker for myocardial injury, remained within normal limits at baseline and after the first two chemotherapy cycles, with levels considered elevated if exceeding 0.09 ng/L. Similarly, B-type natriuretic peptide (BNP) levels were considered elevated if exceeding 100 pg/ml, with significant increases noted after the 6th chemotherapy cycle level. Troponin I levels, a sensitive marker for myocardial injury, remained within normal limits at the baseline and after the first two chemotherapy cycles. A statistically significant elevation in troponin I level was detected after the 4th cycle of chemotherapy (*p* < 0.05). However, in patients who received cardioprotective therapy, including beta-blockers (e.g., carvedilol) and ACE inhibitors (e.g., enalapril), the increase in troponin I levels was significantly attenuated, with elevations averaging 30% lower than those in patients who did not receive such therapy. Similarly, B-type natriuretic peptide (BNP) levels exhibited a more gradual increase in patients undergoing cardioprotective therapy, with significant elevations noted after the 6th cycle, although the increase was approximately 25% less pronounced compared to those not receiving cardioprotective treatment (*p* < 0.01), preceding any notable changes in echocardiographic parameters. This elevation persisted and was more pronounced following the 6th and 8th cycles, consistent with the observed decline in echocardiographic measures of cardiac function, suggesting a correlation between myocardial biochemical injury and functional impairment.

Similarly, B-type natriuretic peptide (BNP), a marker for cardiac strain and heart failure, exhibited a gradual increase throughout the treatment, with significant elevations noted after the 6th chemotherapy cycle (*p* < 0.01). The rise in BNP levels correlated with the echocardiographic evidence of diastolic and systolic dysfunction, further substantiating the developing cardiotoxicity narrative (Table 7).

**Table 7:** Temporal changes in cardiac biomarkers during chemotherapy in breast cancer patients.

Biomarker	Baseline (mean ± SD)	Post 2nd cycle (mean ± SD)	Post 4th cycle (mean ± SD)	Post 6th cycle (mean ± SD)	Post 8th cycle (mean ± SD)	Notes
Troponin I levels (ng/ml)	0.01 ± 0.002	0.01 ± 0.003	0.04 ± 0.006	0.06 ± 0.008	0.07 ± 0.009	Early indicator of myocardial injury; statistically significant elevation after 4th cycle (*p* < 0.05)
B-type natriuretic peptide (BNP, pg/ml)	35 ± 5	37 ± 6	42 ± 7	60 ± 8	70 ± 9	Marker for cardiac strain; significant elevation after 6th cycle (*p* < 0.01)

These biochemical markers, particularly the increase in troponin I and BNP levels, serve as early harbingers of cardiac stress and damage, often preceding detectable changes in cardiac function through imaging. The temporal relationship between these biomarker elevations and changes in echocardiographic parameters underscores the importance of an integrated diagnostic approach, combining biochemical and imaging data, for the early detection and monitoring of chemotherapy-induced cardiotoxicity.

### 3.8 Speckle tracking echocardiography analysis of ventricular strain and strain rate metrics

Employing speckle tracking imaging (STI) technology, we conducted an in-depth analysis of left and right ventricular strain parameters to detect early myocardial deformation indicative of cardiotoxicity. This advanced imaging technique allowed us to quantify the global longitudinal strain (GLS), global longitudinal strain rate (GLSR), global circumferential strain (GCS), global circumferential strain rate (GCSR), global radial strain (GRS), and global radial strain rate (GRSR) for both ventricles, offering a comprehensive assessment of myocardial function and elasticity.

For the left ventricle, baseline measurements of GLS, GLSR, GCS, GCSR, GRS, and GRSR were within the normal ranges, indicating healthy myocardial function prior to chemotherapy initiation. However, as chemotherapy progressed, we observed a significant reduction in GLS and GLSR after the 2nd chemotherapy cycle (*p* < 0.05), suggesting an early impact of chemotherapy on myocardial deformation capabilities. This decline in GLS and GLSR continued to be evident and became more pronounced following the 4th, 6th, and 8th cycles, indicating progressive left ventricular dysfunction.

GCS and GCSR also showed significant reductions, particularly after the 4th chemotherapy cycle (*p* < 0.05), which persisted throughout the subsequent cycles. These changes highlight the circumferential aspect of myocardial deformation affected by chemotherapy. Similarly, GRS and GRSR measurements indicated a decrease in radial function, although these changes were less pronounced than the longitudinal and circumferential strains, suggesting a differential impact of chemotherapy on various components of myocardial deformation.

For the right ventricle, baseline GLS, GLSR, GRS, and GRSR were also within normal limits. However, similar to the left ventricle, there was a noticeable decline in GLS and GLSR after the 4th chemotherapy cycle (*p* < 0.05), which continued to decrease with subsequent cycles. This reduction in right ventricular longitudinal strain and strain rate parameters underscores the global effect of chemotherapy on cardiac function, affecting both ventricles, albeit with varying degrees of severity and timing (Table 8).

**Table 8:** Speckle tracking imaging analysis of myocardial strain parameters throughout chemotherapy treatment.

Parameter (left ventricle)	Baseline (mean ± SD)	Post 2nd cycle (mean ± SD)	Post 4th cycle (mean ± SD)	Post 6th cycle (mean ± SD)	Post 8th cycle (mean ± SD)	Notes
Global longitudinal strain (GLS,%)	−19.5 ± 2.3	−18.0 ± 2.4	−16.5 ± 2.5	−15.8 ± 2.6	−15.2 ± 2.7	Early and progressive dysfunction; significant from 2nd cycle (*p* < 0.05)
Global longitudinal strain rate (GLSR, s^−1^)	−1.2 ± 0.2	−1.1 ± 0.2	−1.0 ± 0.2	−0.9 ± 0.2	−0.8 ± 0.2	Correlates with GLS changes; significant from 2nd cycle (*p* < 0.05)
Global circumferential strain (GCS,%)	−23.0 ± 3.1	−22.5 ± 3.2	−22.0 ± 3.3	−21.5 ± 3.3	−21.0 ± 3.4	Significant reduction from 4th cycle (*p* < 0.05)
Global circumferential strain rate (GCSR, s^−1^)	−1.4 ± 0.2	−1.3 ± 0.2	−1.2 ± 0.2	−1.1 ± 0.2	−1.0 ± 0.2	Parallel to GCS changes; significant from 4th cycle (*p* < 0.05)
Global radial strain (GRS,%)	37.5 ± 4.0	36.8 ± 4.1	36.0 ± 4.2	35.5 ± 4.2	35.0 ± 4.3	Less pronounced changes
Global radial strain rate (GRSR, s^−1^)	2.0 ± 0.3	1.9 ± 0.3	1.8 ± 0.3	1.7 ± 0.3	1.6 ± 0.3	Correlates with GRS changes
Global longitudinal strain (GLS,%)	−22.0 ± 3.0	−21.5 ± 3.1	−20.0 ± 3.2	−19.5 ± 3.3	−19.0 ± 3.4	Significant reduction from 4th cycle (*p* < 0.05)
Global longitudinal strain rate (GLSR, s^−1^)	−1.3 ± 0.2	−1.2 ± 0.2	−1.1 ± 0.2	−1.0 ± 0.2	−0.9 ± 0.2	Correlates with GLS changes; significant from 4th cycle (*p* < 0.05)
Global radial strain (GRS,%)	35.0 ± 4.0	34.5 ± 4.1	34.0 ± 4.2	33.5 ± 4.3	33.0 ± 4.4	Not as pronounced as LV
Global radial strain rate (GRSR, s^−1^)	1.8 ± 0.3	1.7 ± 0.3	1.6 ± 0.3	1.5 ± 0.3	1.4 ± 0.3	Correlates with GRS changes

The comprehensive analysis of these strain parameters provided by STI acquisition has proven invaluable for detecting subtle myocardial deformations that precede overt cardiotoxic effects. The progressive reduction in GLS, GLSR, GCS, GCSR, GRS, and GRSR throughout chemotherapy treatment underscores the importance of advanced imaging modalities such as STI in the early identification and monitoring of chemotherapy-induced cardiotoxicity, offering a window for timely intervention to mitigate cardiac risk in breast cancer patients.

### 3.9 Comparison between multiple groups

In comparing multiple groups across our study, we meticulously analyzed variations in echocardiographic parameters, cardiac biomarkers, and speckle tracking echocardiography (STE)-derived strain metrics to discern the differential impact of chemotherapy on cardiac function.

#### 3.9.1 Echocardiographic parameters

The comparison between groups based on echocardiographic findings revealed a discernible trend towards cardiac function alteration, particularly in diastolic and systolic functions as evidenced by the E/A ratio and LVEF measurements, respectively. While all groups maintained relatively normal LVEF values throughout the initial cycles, a subtle yet significant decline was evident in the groups subjected to anthracycline-based regimens by the 6th and 8th cycles, suggesting a heightened vulnerability to systolic dysfunction. Similarly, the E/A ratio, an indicator of diastolic function, demonstrated a more pronounced decrease in these groups, indicating a differential impact of chemotherapy type on diastolic function.

#### 3.9.2 Cardiac biomarkers

The Analysis of cardiac biomarkers, specifically troponin I and B-type natriuretic peptide (BNP), showed an early biochemical indication of myocardial stress, preceding notable changes in echocardiographic parameters. Groups receiving anthracycline-based chemotherapy exhibited earlier and more significant elevations in both troponin I and BNP levels, aligning with the more severe echocardiographic alterations observed in these groups. This comparison underscores the predictive value of cardiac biomarkers in identifying patients at an increased risk of chemotherapy-induced cardiotoxicity.

#### 3.9.3 Speckle tracking echocardiography (STE) metrics

The STE-derived metrics, including left ventricular global longitudinal strain (GLS) and strain rates (GLSR), circumferential strain (GCS), and radial strain (GRS), offered nuanced insights into myocardial deformation patterns across different groups. The anthracycline-treated groups exhibited earlier and more pronounced reductions in GLS and GLSR, highlighting the sensitivity of these metrics for detecting early myocardial damage. Right ventricular strain metrics, albeit less affected, also showed significant alterations in these groups, suggesting comprehensive ventricular involvement in response to certain chemotherapy agents.

Comparative analysis across multiple groups elucidated the differential impact of chemotherapy regimens on cardiac function, with anthracycline-based treatments associated with more significant cardiotoxic effects, as evidenced by both traditional echocardiographic parameters and advanced STE metrics. The early elevations in cardiac biomarkers further validated the echocardiographic and STE findings, emphasizing the importance of a multimodal approach for monitoring and managing chemotherapy-induced cardiotoxicity. This comparative analysis not only highlights the variable cardiotoxic potential of different chemotherapy agents but also underscores the utility of integrating echocardiographic, biochemical, and STE-derived metrics for a comprehensive assessment of cardiotoxic risk in breast cancer patients undergoing chemotherapy.

### 3.10 Correlation analysis and diagnostic performance of cardiac indicators

#### 3.10.1 Correlation analysis

Our study revealed significant correlations between echocardiographic measures of cardiac function, such as the E/A ratio and LVEF, and STE-derived metrics, particularly GLS. A strong negative correlation was observed between GLS and troponin I levels (r = −0.75, *p* < 0.01), indicating that decreases in GLS were associated with increases in troponin I, highlighting the concordance between mechanical and biochemical markers of myocardial injury. Similarly, B-type natriuretic peptide (BNP) levels showed a moderate negative correlation with GLS (r = −0.65, *p* < 0.01), underscoring the relationship between strain metrics and cardiac stress markers.

#### 3.10.2 ROC curve analysis

The ROC curve analysis was conducted to assess the sensitivity and specificity of GLS, troponin I, and BNP levels in predicting significant cardiotoxicity, defined as a reduction in LVEF below the clinical threshold for cardiotoxicity. GLS demonstrated high diagnostic performance, with an area under the curve (AUC) of 0.92, indicating an excellent ability to discriminate between patients with and without significant cardiotoxicity. Troponin I and BNP also showed good diagnostic performance, with AUC values of 0.85 and 0.83, respectively. The ROC analysis revealed that combining GLS with troponin I levels further enhanced the predictive value for cardiotoxicity, with a combined AUC of 0.95 (Table 9).

**Table 9:** Correlation analysis and ROC curve evaluation of cardiac function metrics and biomarkers in predicting cardiotoxicity.

Metric/analysis	Correlation/findings	Statistical significance	Diagnostic performance (AUC)
GLS and troponin I	Strong negative correlation: decreases in GLS associated with increases in troponin I	r = −0.75, *p* < 0.01	GLS AUC: 0.92
GLS and BNP	Moderate negative correlation: GLS related to BNP levels	r = −0.65, *p* < 0.01	BNP AUC: 0.83
Troponin I	–	–	Troponin I AUC: 0.85
Combined GLS and troponin I	–	–	Combined AUC: 0.95

Correlation analysis and ROC curve evaluation provided a comprehensive understanding of the relationships between the various cardiac health indicators and their collective utility in predicting cardiotoxicity. The strong correlation between mechanical strain metrics and biochemical markers highlights the multifaceted nature of chemotherapy-induced cardiac damage. Furthermore, the ROC curve analysis underscores the superior predictive value of GLS, particularly when combined with troponin I levels, offering a robust approach for the early detection of cardiotoxicity in breast cancer patients undergoing chemotherapy. These findings advocate for an integrated monitoring strategy that leverages both imaging and biochemical markers to identify patients at risk of cardiotoxicity, facilitating timely and targeted interventions to preserve cardiac health.

## 4 Discussion

While our study's comprehensive approach of combining blood checks on different days with echocardiographic assessmentsrequired considerable effort from both patients and healthcare providers, it was essential to capture the dynamic and temporal changes in cardiac function associated with chemotherapy. This intensive monitoring allowed us to detect early signs of cardiotoxicity that might otherwise go unnoticed if the assessments were less frequent. However, we recognize the burden that this approach places on the clinical practice. Future studies should explore optimizing the timing of these assessments to reduce the frequency of visits without compromising the sensitivity of early cardiotoxicity detection. Additionally, integrating these assessments into routine clinical workflows more efficiently could help balance the need for close monitoring with the practicalities of patient care. Although our study provides valuable insights into the early detection of chemotherapy-induced cardiotoxicity, it is important to acknowledge that radial and circumferential global longitudinal strain (GLS) measurements have not yet been fully validated in clinical practice. Although these strain parameters offer a comprehensive view of myocardial deformation and have shown promise in detecting early myocardial dysfunction, their clinical utility remains under investigation. The lack of widespread validation indicates that these parameters should be interpreted with caution, and further research is needed to establish their reliability and clinical significance. Nevertheless, our findings suggest that radial and circumferential GLS may contribute to a more nuanced understanding of myocardial changes during chemotherapy, potentially complementing established global longitudinal strain (GLS) measurements. Future studies should focus on validating these parameters using larger cohorts to confirm their role in clinical practice. The utility of global longitudinal strain (GLS) as a sensitive marker for myocardial deformation, as highlighted in our findings, was reinforced by validation through cardiac MRI in a subset of patients. However, the primary focus of our study was on echocardiographic parameters, particularly GLS, due to their practicality and widespread availability in clinical settings. This emphasis aligns with the goal of developing accessible and reliable diagnostic strategies for early detection of chemotherapy-induced cardiotoxicity in breast cancer patients., including the study by (17), which underscored GLS's predictive value in the context of anthracycline-induced cardiotoxicity. This concurrence reinforces the importance of incorporating advanced strain imaging in routine cardiac monitoring protocols for patients undergoing chemotherapy, offering a more sensitive alternative to traditional echocardiographic measures such as LVEF that may not detect early subclinical changes.

Our findings also highlight the potential benefits of cardioprotective therapy, specifically the use of beta blockers such as carvedilol and ACE inhibitors such as enalapril, in reducing the severity of chemotherapy-induced myocardial injury. This is evidenced by lower elevations in troponin I and BNP levels among patients receiving such interventions, with troponin I levels reduced by an average of 30% and BNP levels reduced by approximately 25% compared to those not on cardioprotective therapy. These results suggest that cardioprotective therapy not only plays a crucial role in reducing clinical cardiotoxicity but also in attenuating biochemical markers of cardiac stress, offering a comprehensive approach to preserving cardiac health in patients undergoing potentially cardiotoxic chemotherapy. These observations align with the existing literature on the protective effects of beta-blockers and ACE inhibitors and underscore the importance of incorporating these therapies into clinical practice, particularly for patients at a high risk of cardiotoxicity. Similar to the patterns observed by (18), elevation in these biomarkers precedes the manifestation of structural cardiac changes, serving as a harbinger of myocardial stress. This underscores the potential of a biomarker-guided approach to complement imaging assessments, enhancing the early identification and stratification of cardiotoxic risk among cancer patients.

Furthermore, the observed correlation between the decrease in GLS and the increase in white blood cell count (from 6.5 × 10⁹/L to 9.0 × 10⁹/L) and neutrophil count (from 3.5 × 10⁹/L to 5.8 × 10⁹/L) suggests a potential link between systemic inflammatory or hematologic responses and myocardial strain alterations. This finding aligns with the existing literature, indicating that systemic stress responses during chemotherapy can affect cardiac function. This correlation underscores the importance of an integrated approach to monitoring, where both cardiac imaging and hematological parameters are assessed together to provide a more comprehensive understanding of a patient's cardiotoxic risk. Future studies should further investigate this relationship to elucidate the underlying mechanisms and determine if changes in blood counts could serve as early indicators of myocardial strain alterations. The integration of GLS with troponin levels, as suggested by our ROC analysis, aligns with the systematic review by (19), which advocated for the combined use of imaging and biomarkers to improve the diagnostic accuracy for cardiotoxicity. This multimodal approach is pivotal for the early detection and timely intervention of cardiotoxicity, potentially allowing the optimization of cancer treatment regimens to mitigate cardiovascular risks.

Furthermore, the need for comparative analyses, as emphasized by (20), to evaluate the cardiotoxic potentials of various chemotherapy agents was addressed in our study through the differential assessment of cardiac function across treatment regimens. This comparative perspective not only enriches our understanding of the cardiotoxic nuances associated with different chemotherapeutic agents but also underscores the importance of personalized treatment planning to balance oncologic efficacy with cardiovascular safety.

In light of these findings, our study underscores the need for a paradigm shift in the management of patients with breast cancer undergoing chemotherapy, advocating for an integrated, multidisciplinary approach that encompasses advanced imaging techniques, biomarker analysis, and a nuanced understanding of the cardiotoxic profiles of chemotherapy regimens. Such an approach not only holds promise for enhancing the early detection of cardiotoxicity, but also for facilitating more informed clinical decision-making, thereby optimizing patient outcomes at the intersection of oncology and cardiology. This integrated monitoring strategy, in concert with evolving treatment paradigms, paves the way for a more holistic and patient-centric model of care, where the dual goals of maximizing cancer treatment efficacy and preserving cardiac health are pursued with equal vigor.

## 5 Conclusion

In conclusion, our study addresses the significant challenge of chemotherapy-induced cardiotoxicity in breast cancer treatment, emphasizing its potential impact on both the effectiveness of cancer therapy and cardiac health of patients. Employing a prospective cohort design, we enrolled breast cancer patients scheduled for potentially cardiotoxic chemotherapy regimens to explore the benefits of an integrated diagnostic approach. Utilizing advanced imaging techniques, such as echocardiography with strain imaging and cardiac MRI, along with serial measurements of cardiac biomarkers, such as high-sensitivity troponins and natriuretic peptides, we aimed to enhance the early detection of cardiotoxic effects. Our analysis revealed that subtle myocardial strain alterations and early biomarker elevations serve as predictive markers for subsequent declines in left ventricular function, providing critical insights before traditional echocardiographic signs of cardiotoxicity become evident. The study's logistic regression analysis underscored the added value of combining biomarker data with advanced imaging findings to identify patients at the highest risk for significant cardiotoxicity. The conclusions drawn from our investigation advocate for a comprehensive diagnostic strategy that integrates detailed imaging assessments with sensitive biomarker analysis, offering a superior method for early detection of chemotherapy-induced cardiotoxicity in breast cancer patients. This proactive approach enables clinicians to tailor cancer therapy with greater precision and carefully balance oncologic efficacy against cardiovascular safety. This highlights the crucial role of a multidisciplinary approach in managing patients undergoing potentially cardiotoxic chemotherapy, ensuring that cancer treatment does not compromise the well-being of the heart. In summary, our study promotes the adoption of an advanced multimodal diagnostic framework that prioritizes early detection and timely intervention, paving the way for personalized medicine to align the goals of effective cancer treatment with the preservation of cardiovascular health.

## Data Availability

The original contributions presented in the study are included in the article/supplementary material, further inquiries can be directed to the corresponding author/s.
