# Corrigendum: Early detection of chemotherapy-induced cardiotoxicity in breast cancer patients: a comprehensive analysis using speckle tracking echocardiography

**DOI:** 10.3389/fcvm.2025.1548119

**Published:** 2025-03-03

**Authors:** Ning Zhang, Na Wang, Yanyan Zhang, Ya Liu, Miaomiao Pei, Gaiqin Liu, Xinle Jia, Xuejia Guo

**Affiliations:** Department of Ultrasound, The First Hospital of Hebei Medical University, Shijiazhuang, China

**Keywords:** breast cancer, chemotherapy-induced cardiotoxicity, echocardiography, cardiac MRI, biomarkers, early detection

A Corrigendum on Early detection of chemotherapy-induced cardiotoxicity in breast cancer patients: a comprehensive analysis using speckle tracking echocardiography By Zhang N, Wang N, Zhang Y, Liu Y, Pei M, Liu G, Jia X and Guo X (2024). Front. Cardiovasc. Med. 11. doi: 10.3389/fcvm.2024.1413827

In the published article there was an error in the order of the authorship. The corresponding author was incorrectly placed first in the list. The correct author list is: Ning Zhang, Na Wang, Yanyan Zhang, Ya Liu, Miaomiao Pei, Gaiqin Liu, Xinle Jia, Xuejia Guo*.

The authors apologize for this error and state that this does not change the scientific conclusions of the article in any way. The original article has been updated.

